# Psychometric properties of the pain anxiety symptom scale among postoperative patients in Amol, Iran

**DOI:** 10.3389/fpsyt.2024.1422346

**Published:** 2024-12-06

**Authors:** Hamid Sharif-Nia, Erika Sivarajan Froelicher, João Marôco, Esmaeil Hoseinzadeh, Sima Hejazi, Reza Fatehi, Poorya Nowrozi, Bita Mohammadi

**Affiliations:** ^1^ Psychosomatic Research Center, Mazandaran University of Medical Sciences, Sari, Iran; ^2^ Department of Nursing, Amol School of Nursing and Midwifery, Mazandaran University of Medical Sciences, Sari, Iran; ^3^ Department of Physiological Nursing, School of Nursing, University of California, San Francisco, San Francisco, CA, United States; ^4^ Department of Epidemiology and Biostatistics, School of Medicine, University of California, San Francisco, San Francisco, CA, United States; ^5^ William James Centre for Research, ISPA - Instituto Universitario, Lisboa, Portugal; ^6^ Department of Nursing, Faculty of Medical Sciences, Islamic Azad University, Gorgan, Iran; ^7^ Addiction and Behavioral Sciences Center, North Khorasan University of Medical Sciences, Bojnurd, Iran; ^8^ Nursing Department, Bojnurd School of Nursing and Midwifery, North Khorasan University of Medical Sciences, Bojnurd, Iran; ^9^ Department of Nursing, Student Research Committee, Mazandaran University of Medical Sciences, Sari, Iran; ^10^ Mazandaran University of Medical Sciences, Sari, Iran

**Keywords:** anxiety, pain, psychometrics, reliability, validity, Iran

## Abstract

**Introduction:**

So far, the psychometric properties of the Persian version of the Pain Anxiety Symptom Scale have not been assessed in Iran. Therefore, this study was conducted to determine the validity and reliability of the Persian version of the Pain Anxiety Symptom Scale among a group of Iranian patients in Amol.

**Methods:**

This methodological study was conducted in 2023 with a sample of 400 postoperative patients from Amol, Iran, selected through convenience sampling. The dataset was divided into two groups of 200 for exploratory and confirmatory factor analyses. Construct validity was assessed using maximum likelihood exploratory factor analysis with Promax rotation, supported by Horn’s parallel analysis and network analysis to visualize item relationships. Confirmatory factor analysis, convergent and discriminant validity was performed on the second dataset. Reliability was evaluated through various statistical measures, including Cronbach’s alpha, McDonald’s omega, average inter-item correlation coefficient, composite reliability, and maximal reliability (MaxR).

**Results:**

Among the 400 participants, the mean age was 44.38 years (SD = 13.49), with 152 (46.1%) being women and 178 (53.9%) men. Most participants (n = 268, 81.2%) had an education level lower than a diploma, and 93 individuals (28.2%) reported a history of surgery. The results of exploratory factor analysis with Promax rotation developed two factors accounting for 66.29% of the variance comprising 15 items. Also, after necessary modifications during confirmatory factor analysis, the final model was approved. As for reliability, the Cronbach’s alpha, composite reliability, and MaxR for all constructs were greater than 0.7, demonstrating good internal consistency and construct reliability.

**Conclusion:**

According to the results, the Persian version of the Pain Anxiety Symptom Scale has a valid structure and acceptable reliability. This scale can be used by health professionals in many ways.

## Introduction

Surgery and anesthesia are special care measures that preserve people’s lives and reduce millions of deaths and diseases worldwide. The need for this care is expected to increase in the coming decades (1). However, they come with consequences. Pain is the most common distressing aspect of surgery (2). Emotional reactions to pain, such as pain-related anxiety, impact the emotional responses to pain (3). One theory of the role of fear and anxiety in pain development is the fear-anxiety avoidance model (4). Pain anxiety plays a crucial role in postoperative recovery, as it can worsen the experience of pain and impede rehabilitation efforts. Studies have shown that patients with high levels of pain-related anxiety are more likely to experience heightened pain levels and longer periods of disability after surgery (5). Surgery is a common source of stress for many patients, often resulting in increased levels of pain and anxiety. Studies have consistently demonstrated that preoperative anxiety can significantly impact postoperative pain levels. Patients who experience high levels of anxiety before surgery are more likely to report higher levels of pain after the procedure, leading to a greater need for pain medication to effectively manage their discomfort (6).

Pain anxiety is an emotional state associated with the anticipation of pain and its negative impacts (7). Pain anxiety is accompanied by cognitive (worry), emotional (fear), behavioral (escape/avoidance), and physiological responses to pain or pain prediction (8). The presence of this anxiety leads to an increase in the pain component of suffering (3). Also, this pain response is predictive of reduced physical activity and disability (9). Patients with pain-related anxiety tend to pay more attention to pain-related stimuli than other patients (4). Many children and adolescents experience anxiety before surgery, which can lead to increased postoperative pain and delayed recovery. Studies have shown that those who are more anxious before surgery are likely to experience more pain afterward, similar to adults (10). Therefore, since postoperative pain is a common occurrence after surgery in adolescents, the likelihood of pain anxiety occurring is also not far-fetched.

Cultural beliefs influence how individuals experience and manage pain and anxiety. People may understate their discomfort due to cultural taboos on mental health, which can impact how they communicate with healthcare providers and their treatment outcomes (11). Cultural norms strongly impact how people express pain. Some cultures promote hiding pain while others encourage vocalizing it. Research shows that different cultural groups have different ways of dealing with pain, with some remaining quiet and others seeking support. It is important to consider these cultural differences when addressing pain in diverse populations (12). In Iranian culture, social norms impact how people show discomfort, so it’s important for healthcare providers to understand these dynamics. Research shows that cultural factors influence how mental health issues, like pain and anxiety, are perceived and communicated in Iran. This underscores the need for culturally sensitive care in addressing psychological and physical recovery (13).

Jeffrey Gray’s Revised Reinforcement Sensitivity Theory (rRST) outlines how the Behavioral Inhibition System (BIS), Behavioral Activation System (BAS), and Fight-Flight-Freeze System (FFFS) interact to influence emotions and behaviors, particularly fear and avoidance. The BIS triggers avoidance behaviors in response to perceived threats, while recent advances highlight the complex relationships among these systems and the subtle effects of rewards and punishments on behavior. Although not widely used in validation studies, rRST improves our understanding of the connections between fear, avoidance, and motivation (14). rRST posits that fear and anxiety are processed differently in the brain. Fear is triggered by clear threats, leading to active avoidance behaviors like fighting or fleeing, while anxiety arises from uncertain situations, promoting passive avoidance and heightened caution. Anxiety involves complex cognitive evaluations of potential, unclear threats, contrasting with fear’s immediate response to obvious dangers (15).

The study shows that preoperative anxiety, influenced by the BIS, can worsen postoperative pain experiences. Patients with high anxiety levels may require more pain medication after surgery (16). Cultural factors also play a role in how individuals perceive and manage pain anxiety during recovery. Healthcare providers can use this knowledge to implement specific interventions to reduce pain anxiety and improve postoperative outcomes (17).

With an understanding of the importance of identifying emotional and cognitive responses to pain in determining the distress individuals endure when facing pain, various tools have been designed by researchers to identify and determine these responses indirectly (3). Examples of these tools include the Cognitive Error Questionnaire (18), Inventory of Negative Thoughts in Response to Pain (19), Pain Cognitions Questionnaire (20), Pain and Impairment Relationship Scale (21), and Fear of Pain Questionnaire (22).

One of the tools designed for measuring emotional responses to pain, namely pain anxiety, is the Pain Anxiety Symptoms Scale (PASS). This scale, which has a full version consisting of 40 items, was first developed by McCracken and colleagues (1992) to assess four dimensions of pain anxiety: cognitive anxiety, escape-avoidance behaviors, fear of pain, and physiological symptoms of anxiety (3, 23, 24). In a study involving chronic pain patients, principal component analysis revealed five factors, including catastrophic thoughts and coping strategies, suggesting that the original model may benefit from further refinement (25). From the 40-item version, a shortened 20-item version was also developed. The original version of this shortened scale has a four-dimensional structure (cognitive anxiety, escape-avoidance behaviors, fear of pain, and physiological symptoms of anxiety). The responses to items are on a six-point scale, where “never” assigns a score of zero and “always” assigns a score of five. The total score on the scale ranges from zero to 100, with higher scores indicating more severe anxiety (23, 26). The PASS has been subjected to both exploratory and confirmatory factor analyses, which have consistently supported its four-factor structure (27). Although in some studies, a two-factor structure has been proposed for this 20-item version (28).

The psychometric properties of this scale have been evaluated in various studies and different populations, such as individuals with non-specific chronic neck pain (4), healthy volunteers, and recipients of physiotherapy services (27, 29), individuals with chronic pain (23, 24, 30), low back pain (31, 32), workers with occupational injuries (33), musculoskeletal chronic pain (9), and various versions have been prepared in Chinese (8, 9), Spanish (28, 30), Arabic (27), Korean (23), and German (31) languages. Overall, the PASS-20 is a reliable scale for assessing pain-related anxiety in various populations, particularly within clinical contexts and nonclinical.

The Persian version of this scale has been evaluated in three studies conducted on individuals with chronic low back pain (32), neck pain (4), and workers with occupational injuries (33) in Iran. The confirmatory factor analysis results for the Persian version of the PASS-20 indicated a good fit with the original model, with factor loadings ranging from 0.70 to 0.92, demonstrating strong item contributions to their respective factors. For instance, items related to fear of pain exhibited the highest loadings, suggesting that these aspects are particularly salient in the context of pain anxiety among Iranian patients. In terms of sociodemographic factors, studies have indicated that pain anxiety levels can vary significantly based on gender, age, and pain intensity. For example, the Persian PASS-20 scores were notably higher in females and individuals with greater pain intensity and disability (4).

Addressing pain anxiety in surgical patients is crucial as it can greatly impact patient outcomes. Research has demonstrated that interventions aimed at reducing pain anxiety can result in lower pain levels and improved recovery in postoperative individuals. Therefore, it is imperative to comprehend the psychometric properties of the PASS specifically within the Iranian patient population. This understanding is essential for developing personalized interventions that effectively tackle this complex issue (29). However, so far, this scale has not been psychometrically evaluated in Persian-speaking adolescents undergoing surgery. Therefore, this study was conducted to determine the psychometric properties of the Persian version of the PASS in adolescents undergoing surgery.

This study aims to assess the psychometric properties of the PASS among surgical Iranian patients. Despite the well-documented importance of pain anxiety in postoperative outcomes, there is a lack of validated instruments specifically tailored for the Iranian population. The key issues addressed in this research include evaluating the reliability and validity of the PASS in this context, examining its factor structure, and determining its utility in predicting pain-related outcomes in surgical patients. The main research question guiding this study is: How reliable and valid is the PASS for assessing pain anxiety among a group of Iranian patients in Amol, and what implications does this have for clinical practice in Iran?

## Methods

This cross-section methodological study was carried out between October to December 2023. Patients from Amol (Amol, Iran) were recruited for this study.

### Inclusion and exclusion criteria

The inclusion criteria for participants in the study were: being at least 18 years old, being able to communicate in Farsi and being literate, volunteering to participate, and being hospitalized in hospital wards after surgery. The exclusion criteria for the study included cognitive impairments that severely hinder understanding or adaptation, as well as the presence of severe mental illnesses that could interfere with the study results, such as schizophrenia. Additionally, individuals with a decreased level of consciousness were excluded, along with those suffering from heart diseases like uncontrolled unstable angina and severe uncontrolled arrhythmia. Participants with limited activity due to severe physical diseases or cerebrovascular conditions were also excluded, as were pregnant individuals and those with cancer or malignancies. Furthermore, other neurological diseases, rheumatoid arthritis, substance use disorder, and dependence were considered exclusionary factors for this study.

MacCallum et al. (1999) recommended a sample size of at least 200 cases for psychometric studies (34). So, we decided to extend an invitation to 400 people due to the necessity of two different samples for construct validity. In this study, a total of 504 postoperative patients were recruited using a convenience sampling method. However, within this framework, participants were randomly selected from those who met the inclusion criteria and were available at our surgical unit during the study period. Following a thorough explanation of the study’s objectives, the participants were given scale to fill out.

### The original version of the scale

This scale is a self-report scale developed by McCracken (1992). The short form of this scale includes 20 items prepared by McCracken and Dhingra and is based on the main 40-item scale of pain anxiety symptoms. Short-form scores range from 0 to 100, and subjects must answer questions on a scale of 0 (never) to 5 (always). The score range is as follows: avoidance (0 to35) such as “ I go immediately to bed when I feel severe pain “, fear evaluation (0 to 40) such as “Pain sensations are terrifying”, physiological response (0 to 25) such as “ Pain makes me nauseous “ and the total score (0 to 100). From 0 to 34 indicates mild pain anxiety. 35 to 67 indicates moderate pain anxiety and 68 to 100 indicates severe pain anxiety. Participants respond to items such as “ I can’t think straight when in pain” and (3).

### Translation

The scale was translated from English to Persian by the established translation protocols (35). Two translators proficient in both English and Persian independently translated the PASS into Persian. An expert panel, consisting of the authors of this article (add initials of those authors here)and two professional translators, carefully reviewed and combined the two translations to produce a Persian version of the PASS. Following this, a Persian-English translator was hired to translate the PASS-P back into English. The panel of experts then reviewed and approved this PASS-P final version.

### Normal distribution, outliers, and missing data

Skewness (± 3) and kurtosis (± 7) were used to investigate the univariate distribution of data individually. Also, multivariate normality distribution was assessed by the Mardia coefficient of multivariate kurtosis (<8). Mahalanobis d-squared (p < 0.001) was used to determine whether there were any multivariate outliers (36). The missing data were assessed using multiple imputations, and exploratory factor analysis used the pairwise deletion method to handle missing data.

### Construct validity

In order to test construct validity, a dataset of 400 cases was divided into two sets of 200 cases each. The first set was used to conduct a Maximum Likelihood Exploratory Factor Analysis with Promax rotation and Kaiser normalization, utilizing SPSS version 27. This analysis helped determine the factor structure by calculating eigenvalues. These eigenvalues represent the amount of variance in each item that is accounted for by the factor. The percentage of total variance explained by each factor was determined by dividing the eigenvalue by the total number of items (37). In simpler terms, researchers used a method called Horns parallel analysis to find the right extraction factors. A method known as Network Analysis (NA) was employed to identify the underlying relationships within the data. This approach produces a visual representation that illustrates the connections among items, focusing on their direct interrelations rather than grouping metric (38). The Kaiser–Meyer–Olkin (KMO) > 0.8 and Bartlett’s test of sphericity to be significant (*p* < 0.001) were referred to ensure the data was relevant and appropriate for performing the factor analysis. Eigenvalues greater than 1, communalities greater than 0.2, and factor loadings greater than 0.3 were considered in determining the factorability of the data (39–41). A communality threshold of 0.2 is often considered acceptable, particularly in exploratory analyses, as it indicates that a factor explains at least 20% of the variance in an item, which can be sufficient for initial assessments (42). Similarly, a factor loading of 0.3 is frequently cited as a minimum cutoff, suggesting a moderate correlation between an item and its underlying factor (43). These thresholds allow us to retain items that meaningfully and significantly contribute to the identified factors while also ensuring that the analysis remains robust.

### Network analysis

NA generates a network plot, which visually displays the number of factors to retain, based on the items that cluster together, and the strength of their relationship (38). NA is estimated by treating items as nodes, connections between them as correlation, and groups of connected nodes as communities using undirected network models. The Graphical Least Absolute Shrinkage Optimization (GLASSO) method is applied to estimate NA, which involves specific steps for analysis (44):

1. Identification of network nodes: The variables identified in the study as network nodes, representing the entities or concepts we wanted to analyze.
2. Construction of the network: The nodes were connected with links, representing the relationships or associations between the variables.
3. Descriptive analysis: tools used in network science were employed to analyze the topology of the network, such as node centrality, clustering, and other measures.
4. Centrality measures: including strength, closeness, and betweenness, were calculated to determine the importance of each node in the network.
5. Clustering analysis was performed to identify groups of nodes with similar characteristics or relationships.
6. Comparison of networks: Networks from different groups or conditions were compared to identify any significant differences in the structure and relationships between the variables (44, 45).

The following centrality and clustering measures were used for the network analysis:

- Betweenness: This measure indicates the extent to which a variable lies on the shortest path between other variables.
- Closeness: Closeness centrality measures how close a variable is to all other variables in the network.
- Strength: Strength centrality reflects the sum of the weights of the ties connected to a variable.
- Expected Influence: This measure assesses the expected impact of a variable on the network based on its centrality measures.
- Barrat: This measure assesses the clustering coefficient of a variable, indicating how interconnected its neighboring variables are.
- Onnela: It reflects the clustering coefficient of a variable, showing the level of clustering around the variable.
- WS: This measure represents the clustering coefficient of a variable based on the Watts-Strogatz model.
- Zhang: It reflects the clustering coefficient of a variable according to Zhang’s method. In this study, the NA was conducted using JASP version 0.19.0.0 (46).

### Confirmatory factor analysis

In the next step, the factor structure obtained from MLEFA was analyzed and confirmed by conducting CFA based on the second random dataset (n = 200) using AMOS version 27. The following model fit indices were used to assess the model fit: Comparative Fit Index (CFI) was > 0.9, Normed Fit Index (NFI) was > 0.9, Tucker-Lewis Index (TLI) was > 0.9, Relative Fit Index (RFI) was > 0.9, and Incremental Fit Index (IFI) was > 0.9; that of Root Mean Square Error of Approximation (RMSEA) was < 0.08; and for Minimum Discrepancy Function divided by degrees of freedom (CMIN/DF) < 3 was considered good (47).

### Convergent and discriminant validity

For convergent validity, composite reliability (CR) should be greater than 0.7, and Average Variance Extracted (AVE) should be greater than 0.5 for each construct. If AVE is less than 0.5, but CR is more than 0.7, the convergent validity can be considered acceptable (48).

Concerning discriminant validity, this study used the heterotrait-monotrait ratio (HTMT) of the correlations criterion, where the HTMT ratio between all constructs should be less than 0.85 to achieve discriminant validity (49).

### Reliability

The Cronbach’s alpha (α), McDonald’s omega (Ω), average inter-item correlation coefficient (AIC), Composite Reliability (CR), and Maximal Reliability (MaxR) were calculated to gauge the internal consistency and construct reliability. If the α, Ω, CR, and MaxR were greater than 0.7 and AIC values of 0.2 to 0.4 were interpreted as acceptable internal consistency (50).

### Pain anxiety symptom score

Descriptive statistics were employed to calculate the mean score of Pain anxiety. Additionally, an independent samples t-test was conducted to evaluate differences between the groups of men and women concerning Pain anxiety.

### Invariance analysis for sex

We conducted an analysis of measurement invariance following the guidelines proposed by (51), using a multi-step procedure to evaluate configural, metric, and scalar invariance across sex groups (male/female). First, we established a baseline model to test configural invariance, ensuring that the factor structure was consistent across groups. Next, we assessed metric invariance by constraining factor loadings to be equal across groups and compared the fit of this model to the configural model. Finally, scalar invariance was tested by constraining both factor loadings and intercepts to be equal across groups. Model fit at each stage was evaluated using the change in comparative fit index (CFI), with a ΔCFI of less than.01 indicating invariance, alongside the chi-square difference test (Δχ²) to examine significant differences in model fit, where non-significant Δχ² values support invariance. Analysis for Δχ² and ΔCFI were done with the lavTestLRT function from the lavaan package (52) for the R statistical System.

### Ethical approval

This study is a research project component with the ethics code IR.MAZUMS.REC.1403.365 has received approval from the Ethics Committee of Mazandaran University of Medical Sciences. The research team is dedicated to adhering to the highest ethical standards throughout the study. We obtained written informed consent from all participants, ensuring their privacy and confidentiality are safeguarded. The study’s aims and procedures have been clearly explained to the participants, and they have been informed of their right to withdraw at any time. We have taken steps to minimize any potential harm to the participants and ensure that the benefits of the study outweigh the risks. The research team is committed to sharing the study’s findings responsibly and ethically, ensuring that the results are used to enhance healthcare practices and outcomes.

## Results

### Demographic characteristics

The mean age of the participants was 44.38 (SD= 13.49) years. Among the participants 152 (46.1%) were women and 178 (53.9%) were men. The mean age of men was 43.01 (SD= 13.47) and women were 45.98 (SD= 13.39). Most people (n= 268, 81.2%) had an education level lower than a diploma. Additionally, 93 individuals (28.2%) reported a history of surgery.

### The results of MLEFA

**Figure 1:**
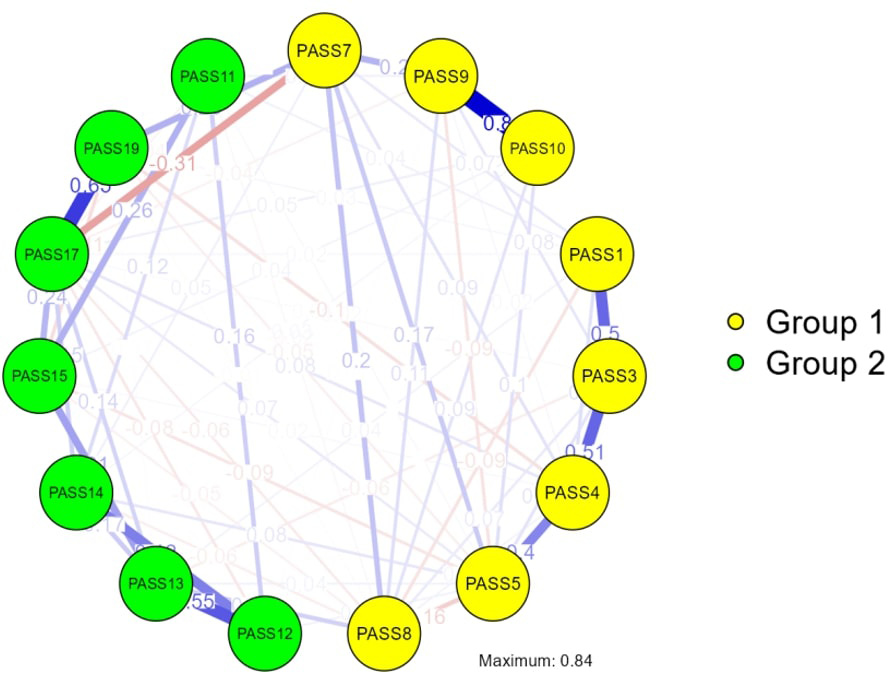
Exploratory graph analysis.

The results of MLEFA with Promax with Kaiser Normalization rotation using the first random dataset (n= 200) developed two factors accounting for 66.29% of the variance comprising 15 items. Item 2, item 6, item 16, item 18, and item 20 were removed from the original version due to communalities of less than 0.2, and factors loading of less than 0.3. Moreover, the results of the KMO (0.927) and Bartlett’s test of sphericity (*p*< 0.001, Chi-squared= 5704.553, *df* = 105) showed the sampling is adequate and appropriate for conducting the factor analysis. The detailed results of the MLEFA are shown in **Table 1**. The results of a network analysis (**Tables 2**, **3**, **Figure 1**):

**Table 1:** The result of MLEFA on the two factors Persian version of Pain Anxiety Symptom Scale (n = 200).

Factor	Items (Q_n_)	Factor loading	h^2^	λ	% Variance
Avoidance behaviours	**Q_9_.** I avoid important activities when I hurt	0.985	0.925	5.967	39.78%
	**Q_10_.** I try to avoid activities that cause pain	0.962	0.916		
	**Q_7_.** I will stop any activity as soon as I sense pain coming on	0.941	0.808		
	**Q_3_.** When I hurt I think about pain constantly	0.912	0.838		
	**Q_4_.** I find it hard to concentrate when I hurt	0.885	0.833		
	**Q_1_.** I can’t think straight when in pain	0.823	0.759		
	**Q_8_.** As soon as pain comes on I take medication to reduce it	0.777	0.564		
	**Q_5_.** I worry when I am in pain	0.539	0.407		
Fear of pain	**Q_13_.** When I feel pain I think I might be seriously ill	0.889	0.867	3.977	26.51%
	**Q_12_.** When I feel pain I am afraid that something terrible will happen	0.876	0.887		
	**Q_14_.** Pain sensations are terrifying	0.849	0.862		
	**Q_15_.** When pain comes on strong I think that I might become paralyzed or more disabled	0.775	0.601		
	**Q_17_.** Pain seems to cause my heart to pound or race	0.675	0.317		
	**Q_11_.** I think that if my pain gets too severe it will never decrease	0.657	0.597		
	**Q_19_.** Pain makes me nauseous	0.460	0.223		

h^2^, Communalities; λ, Eigenvalues

**Table 2:** Centrality measures per item.

Network				
Centrality measures Items	Betweenness	Closeness	Strength	Expected influence
**Q_7_. I will stop any activity as soon as I sense pain coming on**	1.672	1.838	0.489	-0.333
**Q_9_. I avoid important activities when I hurt**	0.257	-0.292	0.786	1.042
**Q_10_. I try to avoid activities that cause pain**	-1.158	-0.748	-0.378	0.846
**Q_1_. I can’t think straight when in pain**	-1.158	-1.528	-1.259	-0.537
**Q_3_. When I hurt I think about pain constantly**	0.129	-0.995	0.198	0.997
**Q_4_. I find it hard to concentrate when I hurt**	0.772	-0.405	0.361	0.881
**Q_5_. I worry when I am in pain**	1.158	0.443	0.478	-0.808
**Q_8_. As soon as pain comes on I take medication to reduce it**	-0.386	0.631	-0.549	-2.144
**Q_12_. When I feel pain I am afraid that something terrible will happen**	-0.643	-0.638	0.016	0.730
**Q_13_. When I feel pain I think I might be seriously ill**	0.772	0.011	0.715	1.463
**Q_14_. Pain sensations are terrifying**	-1.158	-0.348	-0.648	0.256
**Q_15_. When pain comes on strong I think that I might become paralyzed or more disabled**	0.257	0.486	-0.550	-0.803
**Q_17_. Pain seems to cause my heart to pound or race**	1.415	1.556	2.399	0.046
**Q_19_. Pain makes me nauseous**	-0.772	1.159	-0.130	-0.662
**Q_11_. I think that if my pain gets too severe it will never decrease**	-1.158	-1.168	-1.926	-0.976

**Table 3:** Clustering measures per item.

Network				
Clustering measures Items	Barrat	Onnela	WS	Zhang
**Q_1_. I can’t think straight when in pain**	-1.133	-1.252	-0.645	0.995
**Q_3_. When I hurt I think about pain constantly**	0.936	1.426	1.834	-0.333
**Q_4_. I find it hard to concentrate when I hurt**	0.735	1.171	0.801	0.154
**Q_5_. I worry when I am in pain**	-0.662	-0.349	-1.312	-0.354
**Q_7_. I will stop any activity as soon as I sense pain coming on**	1.333	1.558	0.801	0.520
**Q_8_. As soon as pain comes on I take medication to reduce it**	-0.417	-0.736	-1.312	-0.260
**Q_9_. I avoid important activities when I hurt**	-1.019	-0.249	-0.163	-1.495
**Q_10_. I try to avoid activities that cause pain**	0.410	-0.241	1.008	-0.877
**Q_11_. I think that if my pain gets too severe it will never decrease**	-1.444	-1.694	-0.163	-0.996
**Q_12_. When I feel pain I am afraid that something terrible will happen**	-0.367	-1.232	-1.127	0.404
**Q_13_. When I feel pain I think I might be seriously ill**	-0.434	0.251	-0.163	-0.444
**Q_14_. Pain sensations are terrifying**	0.791	1.053	0.801	1.466
**Q_15_. When pain comes on strong I think that I might become paralyzed or more disabled**	0.097	0.377	0.182	-0.616
**Q_17_. Pain seems to cause my heart to pound or race**	-0.822	-0.280	-1.344	-0.469
**Q_19_. Pain makes me nauseous**	1.996	0.196	0.801	2.304

*Centrality and clustering measures*: As shown in **Table 2**, the centrality measures clearly delineate the roles of various items within the network:

*Betweenness*: Items PASS7, PASS5, and PASS17 emerged as crucial connectors within the network. Their high betweenness indicates that they play a pivotal role in linking other symptoms, suggesting they may serve as key targets for interventions aimed at reducing pain anxiety.

*Closeness*: The items PASS7, PASS17, and PASS3 were identified as closely linked to other variables, indicating that they are positioned in a way that allows them to influence other symptoms more directly. This proximity suggests they may be critical in understanding the overall pain anxiety experience.

*Strength*: PASS9, PASS10, and PASS13 demonstrated significant strength, indicating these items have strong direct relationships with other symptoms. Their influence is essential for understanding how pain anxiety manifests in patients.

*Expected Influence*: The expected influence scores for PASS9, PASS10, and PASS13 highlight their potential impact on other symptoms, suggesting that changes in these items could lead to broader changes in pain anxiety levels.

**Table 3** and **Figure 1** illustrates the clustering patterns among key items, providing a visual representation of their interconnections.

*Barrat*: Items PASS19, PASS7, and PASS14 exhibited dense connections, reflecting strong interrelations within the network. This clustering suggests these items may share common underlying factors that contribute to pain anxiety.

*Onnela*: Significant clustering was observed for PASS3, PASS14, and PASS1, indicating that these items form cohesive groups that may reflect similar experiences among patients.

*Weighted Strength (WS)*: The clustering patterns revealed by PASS10, PASS4, and PASS15 suggest notable interdependencies among these symptoms, which could inform targeted therapeutic strategies.

*Zhang*: Finally, strong clustering was noted for PASS19, PASS14, and PASS1, further illustrating the interconnectedness of these symptoms.

### The results of CFA

**Figure 2:**
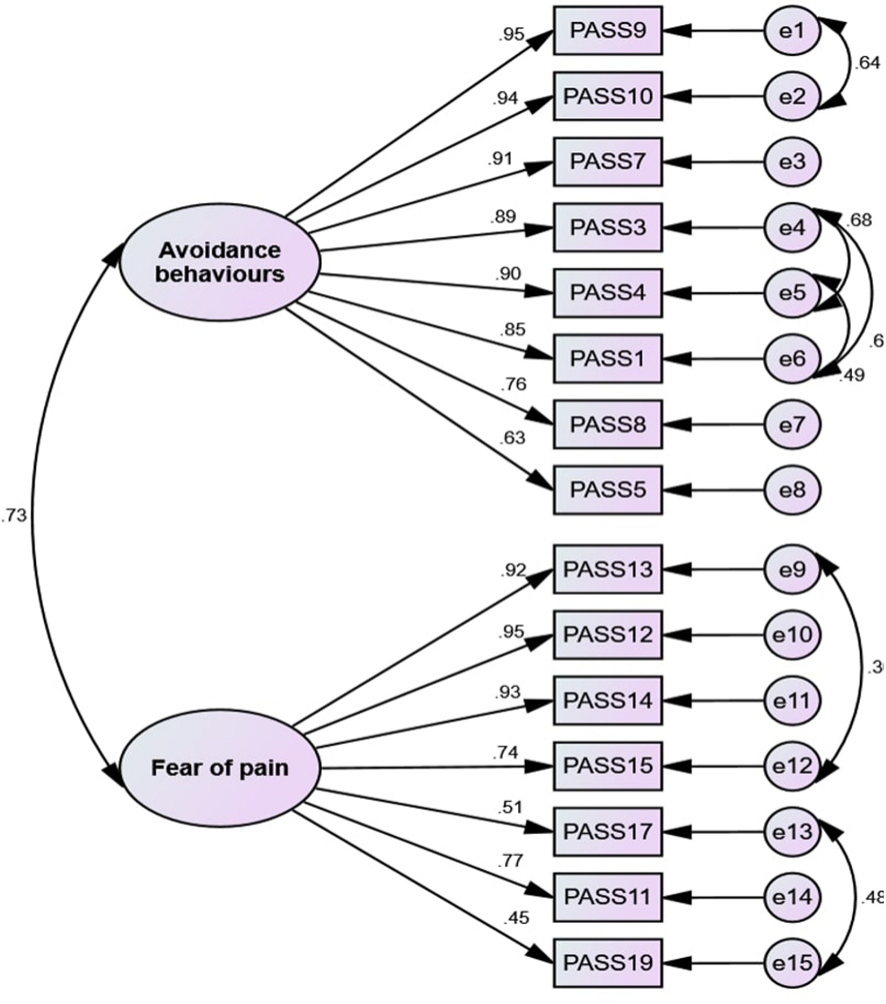
The results of the CFA and factor loadings.

The CFA was conducted to confirm and validate the factor structure obtained from MLEFA using the second random dataset (n= 200). The initial results showed that the data did not fit the model well as evidenced by (*χ* ^2^(83)= 214.916, *p*< 0.001, *χ* ^2^/*df*= 2.589, CFI= 0.977, IFI= 0.977, TLI= 0.971, NFI= 0.963, RFI= 0.953, RMSEA= 0.07. **Figure 2** shows the results of the CFA model.

### Convergent and discriminant validity and reliability

**Table 4** shows the results of the CFA. The results showed that AVE for factors of avoidance behaviors and Fear of pain were greater than 0.5, indicating good convergent validity. The AVE for factors was more than 0.5. factors CR greater than 0.7, it can be concluded that convergent validity for all constructs has been established. As for discriminant validity, the results of the HTMT ratio showed that the correlation between factors of avoidance behaviors and Fear of pain (0.709), was lower than 0.85, demonstrating good discriminant validity for all constructs. As for construct reliability, Cronbach’s alpha, McDonald’s omega, CR, and MaxR for all factors were greater than 0.7, and AIC was more than 0.2, demonstrating good internal consistency and construct reliability (**Table 4**).

**Table 4:** The results of the convergent validity and construct reliability (n= 200).

Factors	α	Ω	AIC	CR	MaxR	AVE
**Avoidance behaviours**	0.953	0.954	0.744	0.957	0.972	0.739
**Fear of pain**	0.913	0.922	0.595	0.909	0.962	0.604

### Invariance analysis for sex

The analysis of invariance was conducted using a sequential model testing approach, which evaluated configural, metric, scalar, and means invariance across groups. Regarding the metric invariance (equal factor loadings between groups), the chi-square difference test (Δχ²= 14.862, Δdf= 13, p= 0.316) was non-significant, indicating that metric invariance was supported. There was no change in CFI (ΔCFI= 0.000), and the RMSEA decreased slightly by 0.005, both further indicating support for metric invariance. Next, for scalar invariance (both factor loadings and intercepts equal between groups), the chi-square difference test again showed no significant deterioration in fit (Δχ²= 8.619, Δdf= 13, p= 0.801), and the CFI increased slightly by 0.001, while RMSEA decreased by 0.005, supporting scalar invariance. These findings indicate that the PASS demonstrated strong invariance across Sex (**Table 5**).

**Table 5:** Analysis of invariance for sex.

Model	Df	AIC	BIC	χ2	Δχ2	Δdf	P[Δχ2(Δdf)≥Δχ2]	CFI	RMSEA	ΔCFI	ΔRMSEA
**Configural**	178	13811.61	14161.12	914.937				0.875	0.158		
**Metric**	191	13799.68	14099.81	929.006	14.862	13.0	0.316	0.875	0.153	<0.001	-0.005
**Scalar**	204	13781.88	14032.62	937.212	8.619	13.0	0.801	0.876	0.148	0.001	-0.005

### Pain anxiety score

In the overall population, the mean score for the Pain Anxiety Symptom Scale was 39.48 (SD= 23.02, 95%CI: 36.98, 41.97). Furthermore, there were no significant (p= 0.538) differences in body esteem scores between men (38.75, SD= 21.96) and women (40.33, SD= 24.26). The mean age of men and women with the Pain Anxiety Symptom score was not significant.

## Discussion

Surgical patients frequently experience preoperative anxiety and postoperative pain. Recent data indicates that 75% of surgical patients continue to experience anxiety even after receiving anti-anxiety interventions. Preoperative anxiety has been linked to increased levels of postoperative pain, with approximately 40% to 65% of patients still reporting moderate to severe pain following surgery (53). The primary aim of the current study was to assess the psychometric characteristics of the PASS in Iranian surgical patients. The findings of this investigation demonstrate that the PASS exhibits a satisfactory factor structure, validity, and reliability.

In interpreting the findings of the PASS among postoperative Iranian patients, it is essential to contextualize these results within the broader literature on pain perception and management. Research indicates that social expectations significantly influence how pain is expressed and perceived, often leading to disparities in treatment outcomes. For instance, women are frequently viewed as more emotional or anxious, which can result in healthcare providers attributing their pain complaints to psychological rather than physiological causes, ultimately affecting their treatment efficacy (54). Furthermore, there exists a well-documented evidence-practice gap in healthcare, where research findings are not effectively translated into clinical practice, often leaving patients without optimal care. This gap underscores the importance of integrating psychometric findings like those from the PASS into clinical protocols to enhance patient management strategies and ensure that all patients receive appropriate pain relief based on their reported symptoms (55).

The results of the study revealed that the Persian version of the PASS included 15 items that were categorized into two subscales: “Avoidance Behaviors” and “Fear of Pain.” These two factors explained 66.29% of the overall variance in pain anxiety levels among Iranian surgical patients. Factor analysis aims to maximize the variance (56). While the original PASS consists of 20 items across four factors, the Promax rotation in this study yielded 15 items distributed into two factors. In another study conducted on patients suffering from acute and chronic spinal pain, a two-factor structure was obtained for this version of the scale (28). In various studies, different factor structures have been obtained for the 20-item version of this scale. Some studies have arrived at the same four-factor structure as the original study in various populations, including individuals attending physiotherapy clinics, those suffering from chronic musculoskeletal pain, and patients with neck pain (4, 9, 27, 57), while others, such as Cho et al. (2010), have identified a three-factor structure in individuals seeking treatment at a pain management clinic (23). Paknejad et al. (2014) also achieved the best model fit for a three-factor model in a population of injured workers (33). This discrepancy may be attributed to cultural variations and the characteristics of the study participants.

The primary factor identified in the PASS was labeled as “Avoidance Behaviors.” Avoidance behaviors are defined as actions that serve to prevent or delay the experience of an unpleasant stimulus. These behaviors can take various forms, ranging from complete avoidance of a particular activity or situation to more subtle behaviors within the situation, such as partial engagement while maintaining some form of avoidance, such as bending down while keeping the back straight. Additionally, avoidance behaviors can also manifest through covert strategies such as distraction and emotional or mental strategies (58). The items associated with this factor highlight the significance of healthcare professionals’ role in recognizing avoidance behaviors in surgical patients. By educating patients on post-operative care and pain management, nurses can help alleviate patients’ anxiety and diminish their avoidance behaviors. This, in turn, enables patients to engage in essential activities independently and reduce their reliance on others.

The second factor identified in the PASS was labeled as “Fear of Pain.” Pain is a subjective experience influenced by various factors, including psychosocial aspects, leading individuals to report and perceive pain differently despite similar injuries. Fear of pain encompasses behavioral, physiological, and verbal reactions to potential or anticipated painful events (59). Many surgical patients may harbor significant apprehension towards experiencing pain postoperatively. Research has demonstrated that preoperative fear of pain can impact the recovery process following surgery (60). Consequently, it is crucial to assess patients’ levels of fear before surgery and actively work toward alleviating their fear of pain. This can be achieved by enhancing patients’ understanding of postoperative pain management through both pharmacological and non-pharmacological interventions, as well as emphasizing the benefits of early mobilization post-surgery to facilitate faster recovery.

In the CFA of the PASS, correlated residuals were specified between several pairs of items, including 9 with 10, 3 with 4, 3 with 1, 4 with 1, 13 with 15, and 17 with 19. Correlated residuals suggest that the corresponding items share some common variance not accounted for by the latent factor, which may be due to similarities in item wording, content, or response formats (61). For instance, items 9 and 10 both refer to the avoidance of activities due to pain, while items 3 and 4 inquire about the fear of pain. These conceptual overlaps may contribute to the correlated errors between these pairs of items. However, it is important to note that the inclusion of correlated residuals should be theoretically justified and not merely used to enhance model fit (62) and can be seen as a potential limitation, as it suggests some shared residual variance among items. To further investigate the impact of correlated errors, future analyses could explore alternative CFA estimation methods that are more suitable for ordinal data, such as robust weighted least squares (WLSMV), which is not available in the AMOS software used in the current study (63). Additionally, the findings indicate that the items in this scale demonstrate strong convergent and divergent validity. Divergent validity reflects the complete separation between constructs, while convergent validity is evident when the elements of a construct are semantically closely related and account for shared variance (56).

Additionally, the internal consistency coefficients of the scale demonstrate that the items within each factor exhibit significant internal correlations, thereby contributing to the clarification and measurement of a broader construct. Essentially, the components of each dimension effectively represent and evaluate a particular concept. Considering the existing cross-cultural gap in health outcomes research (64), the use of the PASS in Persian culture with appropriate cultural variables may facilitate the identification of pain anxiety in surgical patients and provide necessary interventions to reduce it.

Cultural factors play a significant role in shaping pain anxiety and the applicability of the PASS across different contexts. Variations in cultural beliefs and practices can influence how individuals perceive and express pain, as well as their willingness to report it. For instance, some cultures may view stoicism as a virtue, leading individuals to underreport pain or avoid seeking medical help, while others might encourage open expressions of pain as a means of garnering social support (4). Additionally, cultural norms dictate the interpretation of pain-related symptoms, often framing them within spiritual or moral contexts that can affect treatment-seeking behavior (65). Therefore, incorporating cultural competency training for healthcare providers is essential to ensure that the PASS is utilized effectively in diverse populations. This training can help clinicians recognize and adapt to cultural differences in pain expression and anxiety, ultimately improving patient outcomes through more tailored and sensitive care approaches (66).

The results of study demonstrate that the PASS exhibits strong gender invariance among Iranian surgical patients. This finding is consistent with previous research evaluating the psychometric properties of the PASS and its shortened version, the PASS-20, across different populations. A study by Rogers et al. (2020) examined measurement invariance of the PASS-20 across race/ethnicity, sex, and pain in a sample of adults with chronic pain (28). The authors found that the PASS-20 demonstrated strong invariance, indicating that the measure assesses pain-related anxiety similarly across these groups. Similarly, a study by Tashani et al. (2017) investigated the psychometric properties of the Arabic version of the PASS-20 in patients with chronic non-specific neck pain (24). The results supported the four-factor structure of the original PASS-20 and showed good internal consistency and test-retest reliability.

The findings of this study, along with these previous investigations, suggest that the PASS is a robust measure of pain-related anxiety that can be reliably used to assess and compare levels of pain-related fear across gender groups. This is an important characteristic of the scale, as it allows for accurate comparisons and interpretations of PASS scores regardless of the patient’s sex.

Research shows that differences in pain and anxiety symptoms between men and women are influenced by biological, psychological, and social factors. These differences affect how pain is perceived and reported. Estrogen influences how women experience pain by changing their perception and response to pain medications. Hormone fluctuations during the menstrual cycle can also affect pain sensitivity, making women more sensitive to pain at certain times (67). Women tend to be more sensitive to threats, leading to higher levels of anxiety. Research suggests that they have greater startle responses to unpredictable threats, indicating a possible neurological reason for their heightened anxiety compared to men. Women also tend to experience higher levels of pain-related anxiety than men (68).

Women generally experience higher levels of anxiety sensitivity than men. This can lead them to interpret bodily sensations as threatening, which can worsen their experience of pain (69). Women’s higher anxiety levels can also increase their perception of pain. Additionally, women tend to have a more emotional response to fear and anxiety related to pain, which may result in higher levels of fear of pain compared to men. This emotional response can impact coping strategies and the likelihood of seeking treatment for pain (68).

Social expectations influence how men and women express pain, with women often being seen as more emotional or anxious. This can lead to healthcare providers attributing women’s pain to psychological causes rather than physical ones, resulting in less effective treatment. Women may wait longer for treatment and receive less pain relief in emergency settings due to biases that make providers view women’s complaints as less credible or more psychosomatic than men’s (70).

### Limitation

One significant limitation of our study is the absence of a control group, which may impact the generalizability of our findings. While focusing on a specific sample of surgical Iranian patients offers valuable insights into the psychometric properties of the PASS, the lack of a control group hinders our ability to definitively conclude the scale’s performance compared to a broader population or alternative patient groups. Without a control group, we cannot determine whether the pain anxiety symptoms observed are solely due to the surgical context or influenced by other external factors. This limitation is especially crucial in psychometric research, as control groups are vital for establishing baseline measures and ensuring that any observed changes are a result of the intervention or assessment rather than confounding variables.

### Nursing implications

The present study reveals important nursing implications. Nurses should use the PASS for better pain assessment and individualized care planning. Patient education on pain management is crucial, as is interdisciplinary collaboration with other healthcare professionals. Continuous training on the psychological aspects of pain is essential for nurses to provide empathetic and effective care.

In light of the findings from the PASS among postoperative Iranian patients, it is crucial for healthcare professionals to implement targeted interventions that address pain anxiety effectively. One practical strategy is the incorporation of cognitive-behavioral therapy (CBT) techniques, which have been shown to reduce pain-related anxiety and improve coping mechanisms in various patient populations. Additionally, training healthcare providers in effective communication skills can enhance their ability to recognize and validate patients’ pain experiences, fostering a supportive environment that encourages open dialogue about pain anxiety. Furthermore, integrating mindfulness-based interventions into postoperative care can help patients manage anxiety and improve overall pain outcomes. By adopting these strategies, healthcare professionals can better address the psychological aspects of pain, leading to improved patient satisfaction and recovery trajectories.

## Conclusion

The scale comprises two factors encompassing a total of 15 items, explaining 66.29% of the overall variance in pain anxiety among Iranian surgical patients. The findings validate the utility of the Persian version of the PASS as a dependable and valid instrument for evaluating pain anxiety in postoperative individuals. Healthcare practitioners can employ the PASS effectively to instruct patients on methods to alleviate anxiety and pain, consequently promoting accelerated postoperative recovery and reducing the likelihood of complications.

## Data Availability

The raw data supporting the conclusions of this article will be made available by the authors, without undue reservation.
